# Colony Collapse Disorder: A Descriptive Study

**DOI:** 10.1371/journal.pone.0006481

**Published:** 2009-08-03

**Authors:** Dennis vanEngelsdorp, Jay D. Evans, Claude Saegerman, Chris Mullin, Eric Haubruge, Bach Kim Nguyen, Maryann Frazier, Jim Frazier, Diana Cox-Foster, Yanping Chen, Robyn Underwood, David R. Tarpy, Jeffery S. Pettis

**Affiliations:** 1 Pennsylvania Department of Agriculture, Harrisburg, Pennsylvania, United States of America; 2 Department of Entomology, The Pennsylvania State University, University Park, Pennsylvania, United States of America; 3 Department of Infectious and Parasitic Diseases, Epidemiology and Risk analysis applied to the Veterinary Sciences, University of Liege, Liege, Belgium; 4 Department of Functional and Evolutionary Entomology, Gembloux Agricultural University, Gembloux, Belgium; 5 United States Department of Agriculture (USDA) – Agricultural Research Service (ARS) Bee Research Laboratory, Beltsville, Maryland, United States of America; 6 Department of Entomology, North Carolina State University, Raleigh, North Carolina, United States of America; University of Georgia, United States of America

## Abstract

**Background:**

Over the last two winters, there have been large-scale, unexplained losses of managed honey bee (*Apis mellifera* L.) colonies in the United States. In the absence of a known cause, this syndrome was named Colony Collapse Disorder (CCD) because the main trait was a rapid loss of adult worker bees. We initiated a descriptive epizootiological study in order to better characterize CCD and compare risk factor exposure between populations afflicted by and not afflicted by CCD.

**Methods and Principal Findings:**

Of 61 quantified variables (including adult bee physiology, pathogen loads, and pesticide levels), no single measure emerged as a most-likely cause of CCD. Bees in CCD colonies had higher pathogen loads and were co-infected with a greater number of pathogens than control populations, suggesting either an increased exposure to pathogens or a reduced resistance of bees toward pathogens. Levels of the synthetic acaricide coumaphos (used by beekeepers to control the parasitic mite *Varroa destructor*) were higher in control colonies than CCD-affected colonies.

**Conclusions/Significance:**

This is the first comprehensive survey of CCD-affected bee populations that suggests CCD involves an interaction between pathogens and other stress factors. We present evidence that this condition is contagious or the result of exposure to a common risk factor. Potentially important areas for future hypothesis-driven research, including the possible legacy effect of mite parasitism and the role of honey bee resistance to pesticides, are highlighted.

## Introduction

**Figure 1:**
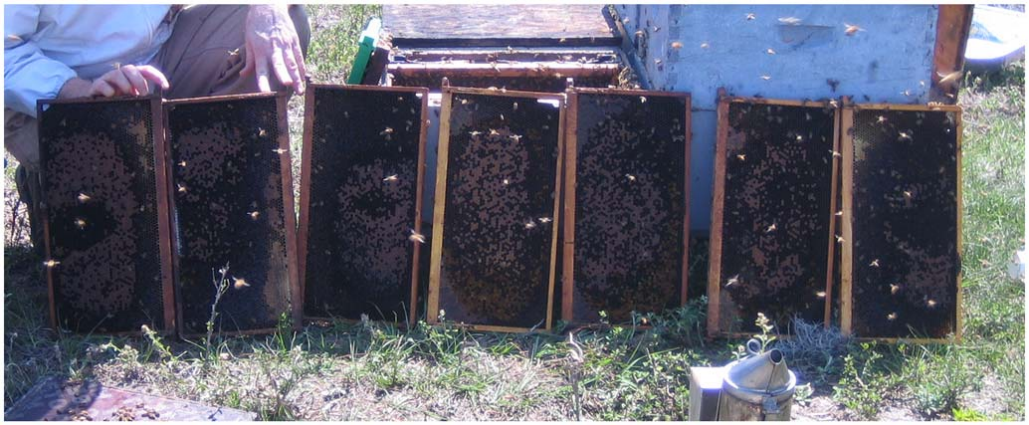
Frames of brood with insufficient bee coverage, indicating the rapid loss of adult bees.

**Figure 2:**
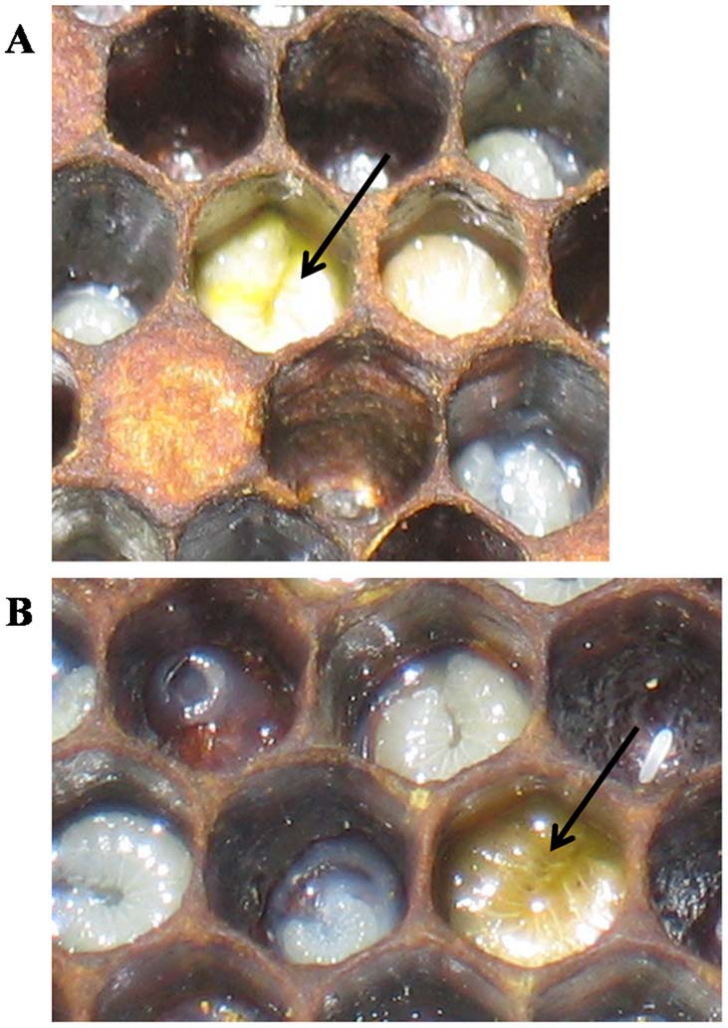
EFB-infected larvae (←) in some CCD-affected colonies were “corn yellow” (A) rather than the typical “beige yellow” (B).

The winter of 2006/2007 witnessed large-scale losses of managed honey bee (*Apis mellifera* L.) colonies in the United States [1]. Those losses continued into the winter of 2007/2008 [2]. In the U.S., a portion of the dead and dying colonies were characterized *post hoc* by a common set of specific symptoms: (1) the rapid loss of adult worker bees from affected colonies as evidenced by weak or dead colonies with excess brood populations relative to adult bee populations (Figure 1); (2) a noticeable lack of dead worker bees both within and surrounding the affected hives; and (3) the delayed invasion of hive pests (e.g., small hive beetles and wax moths) and kleptoparasitism from neighboring honey bee colonies [3]. Subsequently, this syndrome has been termed Colony Collapse Disorder, or CCD.

Large-scale losses are not new to the beekeeping industry; since 1869, there have been at least 18 discrete episodes of unusually high colony mortality documented internationally [4]. In some cases, the descriptions of colony losses were similar to those described above. For example, a condition named “May Disease” occurred in Colorado in 1891 and 1896, where large clusters of bees completely disappeared or significantly declined over a short period of time [5].

Numerous causes of CCD have been proposed, often with little or no supporting data [6]. In an attempt to identify the potential cause(s) of CCD, we conducted an epizootiological survey of CCD-affected and non-affected apiaries. In doing so, we set an operational case definition that we verified by taking measurements of colony populations (brood and adult bees) and collecting samples of adult bees, wax comb, beebread (stored and processed pollen), and brood to test for known honey bee parasites (i.e., varroa mites, *Varroa destructor*, and honey bee tracheal mites, *Acarapis woodi*), pathogens (i.e., bee viruses and *Nosema* spp.), pesticide residues, protein content, genetic lineage, and morphological measurements. The results of an initial metagenomic analysis of some of the samples collected from this effort have already been reported [3].

Broadly defined, epizootiological studies are the study of disease occurrence in animal (in this case honey bee) populations. A primary function of epizootiology is to provide clues as to the etiology of disease [7] as defined in the broadest sense - a departure from perfect health [8]. Descriptive epizootiological studies attempt to elucidate the cause(s) of disease by comparing health and risk factors in “diseased” and “non-diseased” populations [8]. A hallmark of these studies is that they are performed without a specific hypothesis, but they require an ability to classify the surveyed population into “diseased” and “non-diseased” individuals (in this case, colonies) based on a case definition.

Case definitions, especially when little is known about the disease, are often inductive and based on shared readily observable clinical characteristics [9]. Clinical characteristics, such as those used to classify colonies as suffering from CCD, are based on readily available (albeit sometimes broad) characteristics easily identified by “clinicians”, which are often referred to as operational case definitions [8]. The operational case definition of CCD, used in this study, may have a low level of specificity and, thus, runs the risk of misclassifying individual colonies, which in turn can bias results [10]. Some of the characteristics used to define CCD, such as the lack of kleptoparasitism or the rapid loss of adult bees, are not easily quantified yet are readily identified by experienced beekeepers. Such ambiguity often results in skeptics dismissing the described condition as too vague to warrant recognition. The human medical literature, however, is filled with examples of such broadly defined disease (e.g., Gulf war syndrome [11]). Studies based on initially broad operational definitions permit the refinement of the case definition as more knowledge is gained about the condition [8]. Thus, the use of a sensitive, potentially overly inclusive definition is typical when investigating conditions for which the inclusion of suspect cases cannot be validated (e.g., by using laboratory test) and is common when investigating apparently new disease events, particularly when that event may be a new outbreak or epidemic.

The current study aimed to (a) characterize the spatial distribution of strong, weak, and dead colonies in apiaries containing colonies with and without CCD symptoms; (b) quantify and compare measurements among populations suspected to be suffering from CCD with apparently healthy colonies; and (c) gain insight into the cause of CCD. By physically mapping dead and weak colonies within CCD-affected and non-affected apiaries, we determined whether colonies graded with the same “condition” were randomly distributed within apiaries. A non-random distribution (e.g., dead colonies tending to neighbor other dead colonies) would suggest that an infective agent or exposure to a common risk factor may underlie the disorder.

We recognized, up front, that our characterization of CCD is not without bias; many measures, such as quantifying the colony population, are confounded with the overt symptom of CCD (i.e., lack of adult bee population). Other confounding measures are those that quantify colony stress. For example, whole-bee protein levels can serve as an indirect measure of developmental stress [12]. Honey bee larvae require sufficient protein in their brood food to ensure proper development and to optimize their activities during the winter. Farrar [13] showed that the quantity of stored pollen within a colony in the fall is significantly correlated with its spring adult bee population. Measures of mass, total protein, and protein-mass ratio can therefore act as an indirect measure of colony nutrition [13]–[19], parasitism [20]–[23], or both. Differences in these measures may be a consequence (i.e., collapsing colonies are less able to acquire sufficient forage to maintain proper colony health and function) or a contributing cause of the syndrome (e.g., nutritionally stressed colonies are more susceptible to pathogen attack). Another indirect measure of developmental stress is fluctuating asymmetry (FA). FA is defined as random differences in the shape or size of a bilaterally symmetrical character [24], which can be an indicator of individual fitness [25] because organisms exposed to stress during early development show less symmetry than unstressed organisms [26]–[33].

Some factors quantified and compared in this study have known impacts on colony health. Elevated populations of varroa mites, *Nosema* spp., and honey bee tracheal mites (HBTM) are known to damage colonies and may contribute to CCD. Both the HBTM and the varroa mite were introduced into the U.S. in the 1980's and are now widespread. While the number of managed honey bee colonies has been in decline in the U.S. since the 1940's, these mites have been implicated in drastic losses of colonies since their introduction [34]. Similarly, two species of *Nosema* are now widespread across the continental U.S. Historically, nosema disease was thought to be caused by the gut parasite *Nosema apis*, which can be particularly problematic for overwintering colonies [35], [36]. However, a recent survey of historical samples collected from across the U.S. suggests that *N. apis* has been largely displaced by *N. ceranae* over the past decade [37]. While the etiology of *N. ceranae* is poorly understood, it has been implicated with recent large-scale losses experienced by Spanish beekeepers [38], [39]. Other pathogens, including bacteria, fungi, trypanosomes, and viruses, can also significantly impact colony health. An extensive survey of declining and healthy honey bee populations, using metagenomics and targeted polymerase chain reaction (PCR), helped to identify several microbial associates of CCD colonies, the most informative of which was the discistrovirus Israeli acute paralysis virus (IAPV) [4]. In the current study, we assayed colonies for the presence of 12 organisms spanning these different groups using sensitive PCR-based techniques [3], [40], [41]. Moreover, using established protocols testing mitochondrial DNA markers [42], we were able to assign the sampled colonies as either European in origin (Eastern vs. Western) or as African in origin (Northern vs. Southern). If certain mitotypes are found to be more affected by CCD, it could pin-point specific genetic strains of interest for future analyses [43], [44] as well as induce future explorations into unique host-pathogen interactions.

Pesticide exposure is also a risk factor that was quantified in this study. Honey bees can contact and collect pesticides when foraging on crops that have been treated to control pest insects, pathogens, or weeds. In addition, since the late 1980's, U.S. beekeepers have been using miticides within their beehives to control parasitic mites (primarily Varroa mites). A diverse range of pesticides, both grower- and beekeeper-applied, have been detected in hive matrices [45]–[47], and many of these products are known to adversely affect colony health [48]–[50]. Here, we compare both the prevalence and load of different pesticides in the wax, beebread, brood, and adult bees in a subset of CCD-affected and non-affected populations.

## Materials and Methods

### Apiary selection and CCD assessment

In January and February 2007, we selected colonies resident in Florida and California distributed across 13 apiaries owned by 11 different beekeepers. Apiaries were classified as (1) having no colonies with CCD symptoms (‘control’) or (2) having colonies with CCD symptoms (‘CCD’). The operational case definition employed to classify CCD cases verses non-cases were qualitative and made in the field by researchers experienced in clinical bee disease diagnosis. This was as follows (1) the apparent rapid loss of adult worker bees from affected colonies as evidenced by weak or dead colonies with excess brood populations relative to adult bee populations; (2) the noticeable lack of dead worker bees both within and surrounding the hive; and (3) the delayed invasion of hive pests (e.g., small hive beetles and wax moths) and kleptoparasitism from neighboring honey bee colonies. In those CCD colonies where some adult bees remained, there were insufficient numbers of bees to cover the brood, the remaining worker bees appeared young (i.e., adult bees that are unable to fly), and the queen was present. Notably, both dead and weak colonies in CCD apiaries were neither being robbed by bees (despite the lack of available forage in the area as evidenced by the lack of nectar in the comb of strong colonies in the area and by conversations with managing beekeepers) nor were they being attacked by secondary pests (despite the presence of ample honey and beebread in the vacated equipment).

The physical locations of the hives in a subset of the visited apiaries (n = 9) were mapped. We classified these colonies as either ‘alive’ or ‘dead’ (i.e., no live bees) and we classified the living colonies as either ‘weak’ or ‘acceptable’ based on the number of frames of bees (with those having four or fewer frames of bees being considered ‘weak’).

### Colony strength and sample collection

In all, 91 colonies were sampled and used in subsequent analyses. The populations of adult bees and brood were measured in living colonies (n = 79) through the estimation of the total area of comb covered by adult bees or brood [after 51].

At the time of sampling, the presence of overt brood infections (pathogens) was noted. The condition of the quality of the brood pattern was also noted with areas of capped brood containing less than 80% viable brood (as indicated by cells empty of brood) were considered “spotty” while those brood patterns that had less than 20% brood mortality were considered “solid”.

Samples of adult bees (∼150 bees) were removed from a central brood frame, placed into a 50 ml centrifuge tube, and temporarily stored on dry ice before being frozen at –80°C for future processing. A subset of these bees was used for pathogen, protein, and pesticide analyses. An additional sample of ∼320 bees, collected from the same frame, was placed in 75% ethanol in a 125 ml sampling container and used for quantification of varroa mite mean abundance, HBTM prevalence, and *Nosema* spp. spore prevalence and load. Finally, all live and dead (n = 12) colonies had ∼15 cm×15 cm sections of brood comb removed from them, which contained wax and often (but not always) bee brood and beebread. Sampled comb was stored on dry ice before long-term storage at −20°C.

### Physiological and morphological measures

#### Body mass and protein analyses

We used BCA Protein Assay kits (Pierce Scientific, Rockford, IL) to quantify protein content from six separate adult worker honey bees from each of the sampled colonies containing live bees (n = 79). This process uses bicinchoninic acid for the colorimetric detection and quantification of soluble protein (Bradford assay), which indicates the developmental nutrition of bees within a colony during larval feeding [52].

We removed each bee from −80°C storage onto ice and separated its head, gaster (abdomen), and thorax with a razor blade. After the wings and legs were removed from the thorax (because, during shipping, many bees did not have a full complement of appendages), we weighed each body segment to the nearest 0.1 mg using a Metler digital scale. Immediately after weighing, each segment was placed into a separate 1.5 ml microcentrifuge tube on ice. We then added 150 µl, 600 µl, and 500 µl of extraction buffer (1×PBS+0.5% Triton X-100) to the head, abdomen, and thorax tubes, respectively. Each sample was homogenized using a clean plastic pestle, placed on ice for 30 min, and centrifuged at 14,000 g for 5 min. The supernatant was then transferred from each tube to a separate 0.5 ml microcentrifuge tube and frozen at −20°C until further analysis.

We performed the BCA tests by adding 18 µl of 1x phosphate-buffered saline, 2 µl thawed protein extract, and 100 µl BCA working reagent (Pierce Scientific, Rockford, IL) to individual PCR reaction tubes, vortexing and spinning the tubes to homogenize the reagents, and incubating them for 30 min at 37°C on a thermocycler. We then cooled the tubes on ice for 15 min and immediately read their absorbance using a Nanodrop^®^ spectrophotometer. Following the Bradford assay, we calculated the final levels of soluble protein using a standard curve generated from known concentrations of Bovine Serum Albumen.

#### Morphometric measures

From each living colony from which adult bees were sampled into ethanol (n = 76), both forewings from 10 workers were removed and mounted on microscope slides using transparent tape. The wings were then scanned at 600 dpi using a Hewlett Packard ScanJet ADF flatbed scanner. The centroid size of each wing was calculated by determining the relative position (landmark) of 12 vein intersections [after 53] and then calculating the square root of the sum of squared distances between each landmark and the centroid of each forewing [54]. The relative position of each landmark was determined using a script written for UTHSCSA Image Tool software (downloaded from http://ddsdx.uthscsa.edu/dig/itdesc.html) and the resulting data were imported into SAS [55] to automate the centroid-size calculation.

To distinguish between true measures of FA and measurement error, a randomly selected sub-sample of up to 10 bees from 24 colonies (n = 216) had their centroid sizes recalculated from the original scanned image. A two-way ANOVA (repeated measures) revealed that the mean square of the interaction between individual bees and wing side was significantly larger than the mean square of the error term (F = 4.66, df = 215, 432; P<0.0001), suggesting that measurement error was not a significant source of centroid size variation [56].

A simple linear regression was conducted [57] comparing centriod size and FA. As no association was found (F = 0.085, df = 1, 7, P = 0.7714), no correction for scale effect was warranted [56]. Consequently, FA_1_ [58] measures were calculated by determining the absolute difference in centroid size between an individuals left and right wings.

### Risk exploratory variables

#### Macro-parasite and pathogen quantification

The mean abundance of varroa mites (mites per bee, or mpb) was determined by separating mites from the entire sample of bees stored in ethanol by shaking them in soapy water and then counting both the number of mites and bees in the sample [59]–[61]. Thirty of these bees also had their abdomens removed to measure the mean abundance of *Nosema* spp. spores (spores per bee) following Cantwell [62]. Finally, using the methods outlined by Delfinado-Baker [63], the prevalence of honey bee tracheal mites (*Acarapis woodi*) was determined by examining thoracic slices of 16 bees per colony, which is the number suggested for differentiating highly infested colonies (prevalence >30%) and colonies with low infestation (prevalence<10%) [64]. For all of these tests, colonies were additionally classified as being affected or not affected by the parasite or pathogen, regardless of the load.

#### Pathogen analyses

We determined the prevalence (proportion of colonies affected) of several pathogens, including bacteria, trypanosomes, *Nosema* species, and numerous viruses: Acute Bee Paralysis Virus (ABPV), Black Queen Cell Virus (BQCV), Chronic Bee Paralysis Virus (CBPV), Deformed Wing Virus (DWV), Israeli Acute Paralysis Virus (IAPV), Kashmir Bee Virus (KBV), and Sacbrood Virus (SBV). Each pathogen was targeted with a single diagnostic primer [3, 40, 41; Table 1] except IAPV, for which we employed three distinct primer pairs as a means of capturing all members of this diverse lineage. For IAPV, we present relative transcript abundances based on each primer pair separately and an aggregate (arithmetic mean; IAPV_Avg_) from all primer pairs. We extracted total RNA from pooled abdomens of eight worker bees from each colony (n = 76) by grinding abdomens in 1 ml guanidine thiocyanate lysis buffer, pelleting debris, and then extracting RNA from the supernatant using the RNAqueous procedure (Ambion). We then generated cDNA from approximately 500 ng of total RNA using a mixture of poly-dT primers [40] and Superscript II reverse transcriptase (Roche). We carried out quantitative PCR on individual samples and targets using the fluorescent intercalating dye SYBR Green and a Bio-Rad Icycler thermal cycler. We optimized primer pairs for each pathogen target (Table 1) and conducted all PCR reactions using a thermal profile of 3 min at 94°C, followed by 40 cycles of 94°C (30 s), 60°C (30 s), 72°C (30 s), and 78°C (20 s). The 78°C step was used to avoid background signals from potential primer-dimer artifacts. We normalized the estimates of pathogen transcript abundance by the ddC_T_ method [65], using the geometric mean C_T_ value of three honey bee housekeeping genes (actin, RPS5, and mGsT) as a reference for pathogen transcript abundance.

**Table 1:** Quantitative-PCR primers for measuring transcript abundances of honey bee pathogens.

Locus	Forward Primer	Reverse Primer
ABPV	ACCGACAAAGGGTATGATGC	CTTGAGTTTGCGGTGTTCCT
BQCV	TTTAGAGCGAATTCGGAAACA	GGCGTACCGATAAAGATGGA
DWV	GAGATTGAAGCGCATGAACA	TGAATTCAGTGTCGCCCATA
KBV	TGAACGTCGACCTATTGAAAAA	TCGATTTTCCATCAAATGAGC
IAPV_B4SO427	CGAACTTGGTGACTTGAAGG	GCATCAGTCGTCTTCCAGGT
IAPV-F1a	GCGGAGAATATAAGGCTCAG	CTTGCAAGATAAGAAAGGGGG
IAPVpwF16	ACCCCCAACTGCTTTCAACAG	CTGGATATAGTACATTAATGTCCTGC
SBV	GGGTCGAGTGGTACTGGAAA	ACACAACACTCGTGGGTGAC
*N. apis*	CAATATTTTATTGTTCTGCGAGG	TATATTTATTGTATTGCGCGTGCT
*N. ceranae*	CAATATTTTATTATTTTGAGAGA	TATATTTATTGTATTGCGCGTGCA
Trypanosome	CTGAGCTCGCCTTAGGACAC	GTGCAGTTCCGGAGTCTTGT
Bact774	GTAGTCCACGCTGTAAACGATG	GACGGGCGGTGTGTRCA
RPS5	AATTATTTGGTCGCTGGAATTG	TAACGTCCAGCAGAATGTGGTA
Am actin	TTGTATGCCAACACTGTCCTTT	TGGCGCGATGATCTTAATTT
MGST	TTGCTCTGTAAGGTTGTTTTGC	TGTCTGGTTAACTACAAATCCTTCTG

#### Pesticide analyses

Multi-residue pesticide analysis was conducted by the USDA-AMS-NSL at Gastonia, NC, using a modified QuEChERS method [66]. Of the 22 samples of brood comb that contained beebread, 7 had insufficient quantities (<3 g) to analyze on their own, so samples were pooled with other colonies within the same apiary having the same condition (CCD or control) (n = 18). Comb wax, beebread, brood, or adult bees (3 g) were extracted with 27 ml of 44% water, 55% acetonitrile, and 1% glacial acetic acid, after which 6 g of anhydrous magnesium sulfate and 1.5 g anhydrous sodium acetate were added. A 1–2 ml portion of the supernatant was then treated with primary secondary amine, anhydrous magnesium sulfate, and C18 (LC only) or graphitized carbon black (GC only). The resulting supernatant was analyzed by both high-performance liquid chromatography/tandem mass spectroscopy (LC/MS-MS) on a Thermo-Fisher TSQ triple quadrupole MS and gas-liquid chromatography/mass spectroscopy (GC/MS) on an Agilent 5975 triple quadrupole MS for up to171 pesticides and related metabolites [46]. Choices of insecticides, fungicides, and herbicides to analyze were based largely on their frequency of use where bees may be exposed (e.g., in-hive miticides, plant systemics), and their potential for bee toxicity. Limit of detections were in the low part per billion (ppb) range.

#### Genetic analyses

We extracted the DNA from three adult worker bees from each sampled colony (n = 73) using Puregene DNA extraction kits (Gentra systems, Inc.). We then employed an established mitotyping protocol as outlined in Nielsen et al. [42]. This procedure amplifies small (≈1 kb) sections of mitochondrial DNA from the COI and rRNA gene sequences and then subjects them to restriction enzyme digests using *HimfI*, *EcoRI*, and *HincII*. Splicing and banding patterns of the resultant amplified PCR product determined the maternal origin of the bees as either West European (subspecies including *Apis mellifera mellifera*), East European (subspecies including *A. m. ligustica*), North African (*A. m. lamarkii*), or South African (*A. m. scutellata*) after they were electrophoresed on 1.5% agarose gels and visualized with ethidium bromide.

### Statistical analyses

#### Neighboring colony strength ratings

The colonies in all of the mapped CCD apiaries were managed on palletized systems, with either four or six colonies per pallet. Should CCD be caused by an infectious condition or exposure to a common risk factor, we would not expect that colonies in dead or weakened states to be randomly distributed within an apiary but rather be in closer proximity to one another. We tested this hypothesis by comparing the expected and observed frequencies of neighboring colonies (those sharing the same pallet and those with entrances facing in the same direction) with the same or different classifications (dead, weak, or acceptable). As is common in epizootiological studies (e.g. [67]), we examined possible relationships between apparently healthy and diseased colonies by comparing the expected (the number of categorized colonies expected to neighbor one another based on the overall frequency of that condition within an apiary) and observed frequencies of colonies sharing the same strength classification in mapped apiaries using a Chi-square test. The degree (or risk) associated with neighbouring weak or dead colonies in CCD-affected and non-affected apiaries was quantified by calculating odds ratio (95% confidence intervals (logarithmic approximation)). Each neighbor-to-neighbor rating is compared to the reference group as “Adequate – Adequate” neighbor pairings. A *P* value≤0.05 was considered significant.

#### CCD characterization

For statistical purposes, we used two methods to compare CCD and control populations. First, we grouped all colonies within an apiary, and thus compared apiary averages for a given measure in CCD vs. control apiaries. This approach averages the measurements from colonies regardless of whether any particular colony showed signs of collapse and so may include data from colonies not suffering from CCD. However, as sampled apiaries contained colonies that were actively collapsing, colonies graded as “adequately strong” or “control” in CCD apiaries could have been at an early, asymptomatic stage of collapse. Comparing CCD vs. control apiaries reduced the sample size and, consequently, the power of statistical analysis.

The second approach compared adequately strong colonies (control) with colonies that were obviously suffering from CCD (or had presumably died from CCD, such as those that had wax samples analyzed for pesticides; n = 11). While this approach increased the statistical power of analysis, it risked including colonies that were at the early stages of collapse in the control group. We performed and report both types of exploratory comparisons; CCD vs. control populations classified at the apiary- and individual-colony level.

#### Risk explanatory variables analyses

We compared individual- and colony-level measurements between CCD and control apiaries and colonies using Wilcoxon rank sum tests. Nonparametric tests were employed because the basal assumptions of parametric tests (i.e., normality and constant variance) were not satisfied [68]. We assumed that the observations in the two independent samples are representative of the populations of interest. We also compared the incidence (proportion of colonies affected) of the fungal disease chalkbrood (*Ascosphaera apis*), European foulbrood (*Melissococcus pluton*), and spotty brood patterns between the two groups using a Chi-square test or Fisher's exact test when the observed frequency in any cell was less than 5.

Unless otherwise noted, all statistical analyses were carried out using SAS JMP 9.0 [57] When risk factor prevalence data is presented, 95% confidence intervals on the point estimate were calculated by hand to adjust for incident rates based on 100 or fewer cases [8].

## Results

### Colony strength measurements

As the operational case definition for CCD was based, in part, by a clinical assessment that adult bee populations were in rapid decline, differences between non-affected and CCD-affected colony strength measures are not surprising (Table 2 and 3). These results verify that the application of the operational case definition was able to segregate the two populations in a discreet and non-random way.

**Table 2:** Strength and mean physiological and morphometric measurements of bees from colonies (N_t_) located in CCD and control apiaries.

Variable		CCD Apiaries	Mean±S.E.	Median (25th & 75th percentiles)	Control Apiaries	Mean±S.E.	Median (25th & 75th percentiles)	Wilcoxon rank sum test
		N_t_			N_t_			*P*
Strength	Frames of brood	56	2.0±0.24	2.0 (0.3–3.0)	18	1.7±0.45	1.3 (0.8–1.9)	0.46
	Frames of bees	60	5.4±0.68	4.0 (2.0–8.0)	18	7.8±1.26	6.0 (4.0–9.8)	0.02*
	Ratio bees/brood	53	4.7±0.89	2.0 (1.0–4.0)	17	7.5±1.44	4.5 (4.0–10.0)	0.00*
Proteins#	Proteins in the head [A]	60	2.2±0.18	1.3 (1.1–3.4)	18	1.7±0.27	1.3 (1.1–1.7)	0.48
	Proteins in the abdomen [B]	61	12.7±0.82	10.2 (5.6–12.7)	18	10.0±0.98	10.2 (6.0–12.7)	0.21
	Proteins in the thorax [C]	61	4.1±0.87	4.2 (3.4–4.2)	18	4.4±0.18	4.3 (3.9–4.9)	0.19
	Total proteins [D]	60	16.4±0.82	15.4 (12.2–18.4)	18	14.8±1.21	15.4 (10.3–18.3)	0.71
	Mass of the head [E]	60	12.1±0.13	12.1 (11.3–13.1)	18	12.1±0.21	12.1 (11.4–12.9)	0.91
	Mass of the abdomen [F]	61	64.9±1.99	61.6 (55.2–72.3)	18	59.4±3.36	61.1 (47.8–67.4)	0.27
	Mass of the thorax [G]	61	33.5±0.33	33.8 (31.8–35.6)	18	34.1±0.44	34.3 (32.7–35.6)	0.46
	Total mass [H]	60	103.6±2.43	102.5 (92.3–113.4)	18	101.7±3.97	99.9 (91.5–113.2)	0.78
	Ratio [A]/[E]	60	0.10±0.003	0.10 (0.09–0.11)	18	0.11±0.01	0.11 (0.09–0.12)	0.11
	Ratio [B]/[F]	61	0.18±0.007	0.18 (0.15–0.22)	18	0.16±0.01	0.18 (0.15–0.22)	0.22
	Ratio [C]/[G]	61	0.12±0.003	0.12 (0.11–0.14)	18	0.13±0.01	0.13 (0.12–0.14)	0.41
	Ratio [D]/[H]	60	0.15±0.005	0.15 (0.12–0.17)	18	0.14±0.01	0.14 (0.12–0.17)	0.43
Morphological measures	Centroid size	58	59.7±0.79	58.8 (56.6–61.3)	18	60.9±0.73	60.7 (58.4–63.3)	0.08
	FA	58	1.7±0.116	1.48 (1.30–1.98)	18	1.9±0.11	1.9 (1.5–2.2)	0.04*

FA: Fluctuating asymmetry.

# A total of 6 heads or abdomens or thoraces from one colony were used.

* *P*<0.05.

**Table 3:** Strength and mean physiological and morphometric measurements of bees from colonies considered to be normal (control) or affected by CCD (N_t_).

Variable		CCD Colonies	Mean±S.E.	Median (25th & 75th percentiles)	Control Colonies	Mean±S.E.	Median (25th & 75th percentiles)	Wilcoxon rank sum test
		N_t_			N_t_			*P*
Strength	Frames of brood	38	1.5±0.23	1.0 (0.3–3.0)	36	2.4±0.34	1.9 (0.6–3.5)	0.04*
	Frames of bees	39	3.6±0.64	2.0 (1.0–4.5)	39	8.3±0.86	8.0 (4.0–11.00)	0.00*
	Ratio bees/brood	35	4.9±1.15	2.0 (1.0–5.0)	35	6.0±1.00	4.0 (2.3–8.0)	0.05*
Proteins#	Proteins in the head [A]	39	2.2±0.24	1.3 (1.0–3.5)	39	1.9±0.19	1.3 (1.1–2.7)	0.96
	Proteins in the abdomen[B]	39	13.4±1.11	10.9 (9.6–16.6)	40	10.7±0.77	10.3 (6.7–13.4)	0.12
	Proteins in the thorax [C]	39	4.1±0.111	4.2 (3.5–4.6)	40	4.3±0.16	4.2 (3.7–4.8)	0.40
	Total proteins [D]	39	17.1±1.14	15.4 (12.8–18.4)	39	14.9±0.76	15.4 (10.3–18.4)	0.53
	Mass of the head [E]	39	12.1±0.18	11.9 (11.2–13.2)	39	12.2±0.13	12.1 (11.6–12.9)	0.48
	Mass of the abdomen [F]	39	67.2±2.58	63.9 (57.6–72.7)	40	60.2±2.19	58.9 (49.8–70.0)	0.06
	Mass of the thorax [G]	39	33.2±0.41	33.4 (31.7–35.5)	40	34.1±0.34	34.5 (33.0–35.7)	0.12
	Total mass [H]	39	105.6±3.31	102.7 (91.9–116.7)	39	100.8±2.46	101.5 (92.1–112.6)	0.38
	Ratio [A]/[E]	39	0.10±0.004	0.09 (0.08–0.11)	39	0.10±0.003	0.10 (0.09–0.11)	0.20
	Ratio [B]/[F]	39	0.19±0.008	0.18 (0.16–0.23)	40	0.17±0.008	0.18 (0.13–0.20)	0.16
	Ratio [C]/[G]	39	0.12±0.003	0.12 (0.11–0.14)	40	0.13±0.005	0.13 (0.11–0.14)	0.69
	Ratio [D]/[H]	39	0.16±0.006	0.15 (0.14–0.18)	39	0.14±0.005	0.15 (0.11–0.17)	0.22
Morphological measures	Centroid size	36	59.9±1.17	58.8 (56.5–61.1)	40	60.0±0.59	60.0 (56.9–62.4)	0.34
	FA	36	1.5±0.06	1.4 (1.3–1.8)	40	2.0±0.16	1.9 (1.4–2.2)	0.01*

FA: Fluctuating asymmetry.

# A total of 6 heads or abdomens or thoraces from one colony were used.

* *P*<0.05.

N_t_: Number of colonies tested.

### Comparison of apiaries and ratings of neighboring colony strength

CCD-affected apiaries contained 3.5 times the number of dead colonies compared to control apiaries. Similarly, CCD apiaries contained 3.6 times more weak colonies compared to control apiaries (Table 4). In CCD apiaries, neighbouring colonies that were both of adequate strength (‘acceptable’) were 2.3 times less frequent than would have been expected, while neighboring colonies that were both ‘weak’ or both ‘dead’ were approximately 1.3 times more frequent than expected (Table 5). The opposite was true in control apiaries, where adequately strong colonies were 2.6 times more likely to neighbor other colonies of adequate strength. Moreover, the odds ratio demonstrated that in CCD apiaries there was an increased risk of colonies being weak or dead when they neighbored other weak or dead colonies (Table 5). This suggests that CCD is either a contagious condition or results from exposure to a common risk factor.

**Table 4:** Percentage of adequately strong, weak and dead colonies in apiaries containing colonies with symptoms of CCD and apparently healthy (control) apiaries.

Apiary	Location	N	Dead (%)	Weak (%)	Strong (%)
CCD	FL	66	18.1	39.4	42.2
	FL	88	30.6	69.3	0.0
	FL	200	41.0	47.0	12.0
	CA	76	7.9	42.1	50.0
	CA	28	25.0	57.1	17.9
	CA	48	20.8	35.4	43.8
Subtotal		506	28.4	48.6	22.9
Control	FL	64	0	0	100
	CA	34	23.4	38.2	38.2
	CA	88	7.9	13.6	78.4
Subtotal		186	8.1	13.4	78.5

**Table 5:** Observed and expected frequencies of neighboring colonies with similar or different strength ratings in CCD and control apiaries.

Strength Rating		CCD (N = 6)		Control (N = 3)		OR (95% CI)#
Colony 1	Colony 2	Observed	Expected	Observed	Expected	
Adequate	Adequate	28	65	60	23	–
Adequate	Weak	26	26	9	9	5.98 (2.52–14.2)*
Adequate	Dead	15	14	4	5	7.38 (2.36–23.1)*
Weak	Weak	59	44	0	15	255 (15.2–4273)*
Weak	Dead	64	50	3	17	39.5 (12.3–126.5)*
Dead	Dead	25	18	0	7	109.3 (6.42–185.9)*

* *P*<0.05.

# OR: odds ratio; CI: confidence interval.

### Comparison of protein and mass measurements

None of the measurements of soluble protein, mass, or protein-to-mass ratio were different when colonies from CCD apiaries were compared to colonies from control apiaries (Wilcoxon rank sum test; *P*>0.10; Table 2). Similarly, no measures of mass, soluble protein, or protein-to-mass ratio differed between the two types of colonies (Wilcoxon rank sum test; *P*>0.06; Table 3).

### Comparison of morphometric measurements

The average forewing centroid size in bees from colonies sampled in CCD apiaries was no different than bees from colonies sampled in control apiaries (*P* = 0.08). In contrast, a comparison of the absolute difference between the centroid size in right and left wings (FA_1_) revealed that bees from colonies in CCD apiaries were more symmetrical than those in control apiaries (Wilcoxon rank sum test; *P* = 0.04; Table 2).

Similarly, the average centroid size in bees sampled from CCD and control colonies was not different (*P* = 0.34). Bees from CCD colonies, however, were more symmetrical than those in control colonies (Wilcoxon rank sum test; *P* = 0.01; Table 3).

### Comparison of overt signs of disease and brood pattern

Six percent of colonies from CCD apiaries had clinical infections of chalkbrood disease (CB) and 8% had clinical infections of European foulbrood (EFB; Table 6). While none of the colonies in control apiaries had clinical infections with these common brood diseases, the incidence of colonies affected did not differ significantly between apiary types (Fisher's exact test: *P*>0.50). Fifty-five percent of colonies from CCD apiaries had spotty brood patterns, which was not different than the 43% of colonies in control apiaries that had the same condition (*P* = 0.41).

**Table 6:** Parasite and pathogen loads of bees from colonies (N_t_) located in CCD and control apiaries.

Variable		CCD Apiaries		Load		Control Apiaries		Load		Prevalence		Load (Wilcoxon rank sum test)
		N_t_	Prevalence** (95% CI)	Mean±S.E.	Median (25th & 75th percentiles)	N_t_	Prevalence** (95%CI)	Mean±S.E.	Median (25th & 75th percentiles)	χ^2^ *	*P*	*P*
Brood condition	Spotty Brood	49	55 (41–73)			14	43 (23–72)			0.66	0.41	
	Chalkbrood	51	6 (4–8)			17	0				0.56	
	European foulbrood	51	8 (6–11)			17	0				0.57	
Parasites	Varroa†	51	64 (48–84)	0.086±0.0284	0.007 (0–0.038)	17	53 (31–84)	0.020±0.014	0.003 (0–0.014)	0.74	0.39	0.24
	HBTM‡	51	14 (10–18)	1±0.6	0 (0–0)	17	43(25–69)	8±2.9	0 (0–16)	6.41	0.01*	0.01*
	*Nosema♠*	51	55 (41–72)	1.82±0.486	0.7 (0.0–1.80)	17	35 (20–56)	0.34±0.201	0.0 (0.0–0.15)	1.96	0.16	0.09
Pathogens 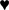	ABPV	58	45 (34–58)	4.4±0.86	0.0 (0.0–7.5)	18	33 (20–52)	2.3±0.90	0.0 (0.0–5.20)	0.75	0.38	0.29
	Bacteria	58	93 (70–100)	12.7±0.80	13.4 (9.1–18.0)	18	100 (59–100)	13.3±1.25	14.2 (7.7–18.0)		0.57	0.86
	BQCV	58	72 (54–93)	9.6±1.02	10.2 (0–13.8)	18	78 (46–100)	7.7±1.67	5.5 (1.1–15.4)		0.77	0.40
	CBPV	58	33 (25–43)	2.4±0.54	0.0 (0.0–3.8)	18	50 (30–79)	1.5±0.42	0.5 (0–3.27)	1.75	0.19	0.66
	DWV	58	44 (34–57)	5.7±0.99	0.0 (0.0–10.1)	18	66 (39–100)	5.6±1.21	5.50 (0–8.5)	2.62	0.11	0.56
	IAPVF1a	58	22 (17–28)	2.0±0.56	0.0 (0.0–0.0)	18	17 (10–27)	1.6±1.06	0.0 (0.0–0.0)		0.74	0.63
	IAPVpw1617	58	16 (12–21)	1.8±0.62	0.0 (0.0–0.0)	18	6 (3–9)	1.2±1.15	0.0 (0.0–0.0)		0.43	0.32
	IAPV_B4SO427	58	10 (8–13)	1.5±0.60	0.0 (0.0–0.0)	18	6 (3–9)	1.1±1.09	0.0 (0.0–0.0)		1	0.58
	IAPVAvg	58	28 (21–36)	1.8±0.52	0.0 (0.0–1.2)	18	17 (10–27)	1.3±1.03	0.0 (0.0–0.0)		0.53	0.36
	KBV	58	29 (22–37)	3.0±0.76	0.0 (0.0–4.8)	18	2 (1–3)	0.7±0.53	0.0 (0.0–0.0)		0.21	0.11
	SBV	58	16 (12–21)	0.8±0.29	0.0 (0.0–0.0)	18	28 (16–44)	2.0±0.84	0.0 (0.0–4.5)	1.37	0.24	0.21
	*Nosema ceraneae*	58	47 (36–61)	5.8±1.00	0.0 (0.0–12.9)	18	72 (42–100)	6.0±1.60	0.0 (0.0–14.6)	3.63	0.06	0.31
	*Nosema apis*	58	28 (21–36)	3.5±0.83	0.0 (0.0–6.47)	18	11 (7–17)	0.09±0.07	0.0 (0. 0–0.0)		0.21	0.09
	Trypanasomes	58	76 (58–98)	8.3±0.84	8.8 (0.0–13.6)	18	94 (56–100)	10.6±1.54	8.17 (5.5–17.7)		0.10	0.26

* Where no statistic is presented, Fisher's exact test was used as some cells had fewer than 5 responses.

** % of colonies infected with organism.

† Load = mean abundance (number of varroa mites per bee) in colonies.

‡ Load = the percentage of bees infested with HBTM per colonies.

♠Load = mean abundance (number of spores per bee (×10^6^)) in colonies.

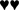
All pathogen loads are scaled relative to the geometric mean of honey bee housekeeping genes RPS5, MGsT, and actin [37].

Colonies suffering from CCD did not have a higher incidence rate of either CB or EFB, nor did they have a greater incidence of poor brood patterns when compared to colonies not apparently suffering from CCD (*P*>0.35; Table 7).

**Table 7:** Parasite and pathogen loads of bees from colonies considered to normal (control) or affected by CCD (N_t_).

Variable		CCD Colonies		Load		Control Colonies		Load		Prevalence		Load (Wilcoxon rank sum test)
		N_t_	Prevalence **(95% CI)	Mean±S.E.	Median (25th & 75th percentiles)	N_t_	Prevalence **(95% CI)	Mean±S.E.	Median (25th & 75th percentiles)	X^2^ *	*P*	*P*
Brood condition	Spotty Brood	35	60 (42–83)			27	52 (34–76)			0.04	0.85	
	Chalkbrood	38	5 (4–7)			39	3 (2–4)				0.62	
	European foulbrood	38	8 (6–11)			39	3 (2–4)				0.36	
Parasites	Varroa†	32	53 (36–75)	0.054±0.020	0.002 (0.0–0.037)	36	70 (49–97)	0.084±0.0372	0.007 (0.0–0.0 29)	1.91	0.17	0.37
	HBTM‡	32	19 (13–27)	0±0.0	0 (0–0)	36	22 (15–30)	10±2	0 (0–0)	0.13	0.72	0.44
	*Nosema♠*	32	63 (43–89)	1.9±0.59	0.1 (0.0–1.9)	36	39 (27–54)	1.0±0.47	0.0 (0.0–0.3)	3.79	0.05*	0.06
Pathogens 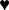	ABPV	38	47 (33–65)	5.1±1.23	0.0 (0.0–8.7)	38	37 (26–51)	2.6±0.62	0.0 (0.0–6.1)	0.86	0.35	0.22
	Bacteria	38	97 (69–100)	12.9±0.91	13.6 (9.2–17.6)	38	92 (65–100)	12.8±0.98	14.1 (8.4–18.0)		0.61	1.00
	BQCV	38	79 (56–100)	10.8±1.31	11.6 (3.5–14.6)	38	68 (48–93)	7.4±1.09	6.3 (0.0–13.7)	1.09	0.30	0.07
	CBPV	38	37 (26–51)	2.6±0.70	0.0 (0.0–3.8)	38	37 (26–51)	1.9±0.51	0.0 (0.0–3.3)	0.00	1.00	0.73
	DWV	38	61 (43–84)	7.7±1.3	6.5 (0.0–12.9)	38	40 (28–55)	3.6±0.86	0.0 (0.0–7.0)	3.37	0.07	0.02*
	IAPVF1a	38	24 (17–33)	2.1±0.69	0.0 (0.0–0.8)	38	18 (13–25)	1.7±0.71	0.0 (0.0–0.0)	0.32	0.57	0.57
	IAPVpw1617	38	16 (11–22)	2.0±0.82	0.0 (0.0–0.0)	38	11 (8–15)	1.3±0.71	0.0 (0.0–0.0)		0.74	0.48
	IAPV_B4SO427	38	11 (8–15)	1.6±0.80	0.0 (0.0–0.0)	38	8 (6–11)	1.2±0.69	0.0 (0.0–0.0)		1.00	0.67
	IAPVAvg	38	29 (21–40)	1.9±0.68	0.0 (0.0–1.5)	38	21 (15–29)	1.4±0.63	0.0 (0.0–0.0)	0.63	0.43	0.42
	KBV	38	42 (30–58)	4.4±1.10	0.0 (0.0–7.0)	38	8 (6–11)	0.5±0.29	0.0 (0.0–0)		0.00*	0.00*
	SBV	38	18 (13–25)	1.1±0.41	0.0 (0.0–0.0)	38	18 (13–25)	1.1±0.44	0.0 (0.0–0.0)	0.00	1.00	0.98
	*Nosema ceranae*	38	55 (39–76)	6.9±1.30	2.3 (0.0–13.8)	38	50 (35–67)	4.8±1.09	0.7 (0.0–9.8)	0.21	0.65	0.34
	*Nosema apis*	38	29 (21–40)	3.5±1.03	0.0 (0.0–6.5)	38	18 (13–25)	1.8±0.80	0.0 (0.0–0.0)	1.16	0.28	0.25
	Trypanosomes	38	82 (58–100)	9.6±1.00	9.8 (5.0–14.1)	38	79 (56–100)	8.2±1.09	7.0 (1.1–13.1)	0.08	0.77	0.31

* Where no statistic is presented, Fisher's exact test was used as some cells had fewer than 5 responses.

** % of colonies infected with organism.

† Load = mean abundance (number of varroa mites per bee) in colonies.

‡ Load = the percentage of bees infested with HBTM per colonies.

♠Load = mean abundance (number of spores per bee (×10^6^)) in colonies.

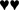
All pathogen loads are scaled relative to the geometric mean of honey bee housekeeping genes RPS5, MGsT, and actin [37].

It is of interest to note that EFB-infected larvae found in one apiary suffering from CCD were distinctly corn-yellow in appearance (Figure 2A) as opposed to the usual beige appearance of infected larvae (Figure 2B). Microscopic examination of smears from these samples revealed nearly pure cultures of EFB's causal agent *Melissococcus pluton*. This is unusual, as EFB smears usually reveal high levels of opportunistic bacteria such as *Paenibacilus alvei, Brevibacillus laterosporus*, and *Enterococcus faecalis* with little or no evidence of the causal agent *M. pluton* [60].

### Comparison of macro-parasite and pathogen prevalence and load

Neither the proportion of colonies affected nor the mean abundance of varroa mites or *Nosema* spp. spores differed between CCD apiaries and control apiaries (*P*>0.05; Table 6). HBTM infection was more than three times as prevalent in control apiaries as compared to CCD apiaries (43% vs. 14% of colonies affected, respectively; *χ^2^* = 6.41, *P* = 0.01; Table 6). The mean prevalence of HBTM in bees from infected colonies was higher in control apiaries than CCD apiaries (8% vs. 1%, respectively; *χ^2^* = 7.71, *P* = 0.01; Table 6).

Neither the prevalence of colonies with varroa mites, *Nosema* spp. spores, or HBTM, nor the load of infection for these macro parasites/pathogens differed between CCD and control colonies (*P*>0.05; Table 7).

### Comparison of pathogen prevalence

None of the screened pathogens showed higher prevalence or load in colonies from CCD apiaries when compared to colonies from control apiaries (Table 6).

Kashmir Bee Virus (KBV) was more prevalent in colonies suffering from CCD as compared to control colonies (42% vs. 8%, respectively; Fisher's exact test *P* = 0001; Table 7). KBV virus titers were higher in CCD colonies when compared to control colonies (*P* = 0.01; Table 7).

Overall, 55% of CCD colonies were infected with 3 or more viruses as compared to 28% of control colonies (Table 8: *χ^2^* = 5.4, P = 0.02). Both *Nosema* species were equally prevalent in CCD and control colonies (Table 7). However, 34% of CCD colonies were found to be co-infected with both *Nosema* species as compared to 13% of control colonies (Fisher's exact test, *P* = 0.05).

**Table 8:** Percentage of Control and CCD colonies infected with Y or more viruses.

Colony classification	n				Percentage (%)			
		Y	1	2	3	4	5	
Control			81.6	60.5	28.9	15.8	7.9	
CCD			84.2	71.1	55.3	31.6	23.7	
		X^2^	0.09	0.94	5.4	2.6	Fisher's	
		P	0.76	0.33	0.02	0.10	0.05	

CCD colonies were co-infected with a greater number of known pathogenic organisms (viruses and *Nosema* species) than control colonies (4.34±0.37 vs. 3.0±0.37, respectively; Wilcoxon rank sum test *P* = 0.026).

### Comparison of pesticide prevalence and residue levels

In all, 50 different pesticide residues and their metabolites were found in the 70 wax samples tested, 20 were found in the 18 pollen (beebread) samples tested, 5 in the 24 brood sampled tested, and 28 in the 16 adult bees tested.

There are some notable constraints with this pesticide data set. The number of beebread and adult-bee samples in control apiaries was low. This was largely a result of insufficient amounts of pollen collected from CCD-affected colonies (n = 7), leading to combining colony samples to obtain a sufficient quantity for analysis (n = 3). After adult bees had been distributed for protein and pathogen analysis, there was only one adult bee sample from a colony in a control apiary available for pesticide analysis. Another issue is that pesticides and metabolites were added to the screen as they became identified within samples. Because the beebread samples were analyzed earlier than the adult bee or brood samples, potentially important pesticides (such as chlorothalonil, amitraz metabolites, and the coumaphos metabolite, chlorferone) were left out of the former but not the latter analyses. Also, a majority of the wax samples were not analyzed for amitraz metabolites, the fungicides boscalid and iprodione, and the coumaphos metabolites chlorferone, coumaphos oxon, and potasan. Where only some of the samples in a given matrix were analyzed for coumaphos metabolites, only coumaphos (and not ‘total coumaphos’ levels - coumaphos plus metabolites) were compared. Lastly, a lack of detection of some chemicals does not necessarily rule out potential exposure. Chemicals that metabolize or break down quickly may have been removed from the various matrixes tested. Alternatively, some chemicals may have been consumed (in the case of beebread) before samples were collected.

There were no differences in the mean number of pesticides detected in the wax of colonies from CCD apiaries (5.96±0.63) compared to colonies from control apiaries (4.87±0.48; *χ^2^* = 0.125, *P* = 0.72). Similarly, there were no differences in the number of detections in beebread (CCD: 4.18±0.62 vs. control: 7.50±0.62; *χ^2^* = 1.83, *P* = 0.175) or brood (CCD: 2.15±0.08 vs. control: 2.00±0.00; *χ^2^* = 0.65, *P* = 0.42).

None of the pesticides detected in more than 20% of the samples in a given matrix was more prevalent in CCD apiaries than in control apiaries (Table 9). There were, however, higher levels of coumaphos in the wax of control apiaries than was detected in CCD apiaries (Wilcoxon rank sum test, *P* = 0.05, Table 9).

**Table 9:** The pesticide residue prevalence and load in wax, beebread, and brood from colonies (N_t_) located in CCD and control apiaries.

Matrix	Chemical	CCD Apiaries		Load		Control Apiaries		Load		Prevalence (Fisher's exact test)	Load (Wilcoxon rank sum test)
		N_t_	Prevalence (%)	Mean±S.E.	Median (25th & 75th percentiles)	N_t_	Prevalence (%)	Mean±S.E.	Median (25th & 75th percentiles)	*P*	*P*
Wax	Boscalid	30	40 (27–57)	29.7±8.46	0.0 (0.0–0.0)	5	0			0.14	0.09
	Chlorothalonil	62	33 (25–42)	24.0±9.11	0.0 (0.0–7.75)	8	13(7–27)	4.9±1.72	0.0 (0.0–0.0)	0.42	0.25
	Chlorpyrifos	62	71 (54–91)	6.3±1.23	4.1 (0.0–7.5)	8	100 (43–100)	5.8±0.64	6.4 (4.1–7.4)	0.11	0.20
	Coumaphos	62	100 (77–100)	3373±552	1750 (719–4085)	8	100 (43–100)	6398±1815	6050 (2110–8992)	1.00	0.05^*^
	Dicofol	62	17 (13–22)	4.1±3.33	0.0 (0.0–0.0)	8	0			0.34	0.20
	Endosulfan	62	25 (19–32)	4.4±2.16	0.0 (0.0–0.33)	8	13(7–27)	0.6±0.55	0.0 (0.0–0.0)	0.67	0.42
	Esfenvalerate	62	13 (10–17)	1.7±0.97	0.0 (0.0–0.0)	8	38(16–75)	1.0±0.52	0.0 (0.0–2.10)	0.11	0.09
	Fluvalinate	62	100 (77–100)	12508±1718	8530 (2452–15608)	8	100 (43–100)	41737±23748	19800 (6510–42575)	1.00	0.06
	Iprodine	35	21 (15–30)	48.9±20.97	0.0 (0.0–0.0)	5	0			0.56	0.26
Beebread	Atrazine	16	32 (18–52)	8.4±16.61	0.0 (0.0–4.57)	2	0	0.0±0.0	0.0 (0.0–0.0)	1.00	0.37
	Chlorpyrifos	16	88 (50–100)	1.8±0.74	0.65 (0.32–1.75)	2	0	0.8±0.05	0.8 (0.7–0.8)	1.00	0.62
	Coumaphos	16	50(29–81)	18.5±7.7	2.1 (0.0–26.3)	2	50 (6–100)	3.6±3.6	0.0 (0.0–4.5)	1.00	0.89
	Dicofol	16	19 (11–31)	0.2±0.49	0.0 (0.0–0.0)	2	100 (12–100)	0.6±0.15	0.0 (0.0–0.7)	0.07	0.08
	Endosulfan1	16	32 (18–52)	0.2±0.38	0.0 (0.0–56)	2	50 (6–100)	0.4±0.4	0.4 (0.0–0.8)	1.00	0.50
	Fenpropathrin	16	50 (29–81)	1.0±0.30	0.4 (0.0–1.6)	2	100 (12–100)	1.5±0.70	1.5 (0.8–2.2)	0.47	0.30
	Fluvalinate	16	94 (54–100)	276±162.5	76 (8–270)	2	100 (12–100)	68±56	68 (12–124)	1.00	0.78
	Malathion	16	19 (11–31)	0.7±0.48	0.0 (0.0–0.0)	2	50 (6–100)	1.8±1.8	1.8 (0–3.6)	0.41	0.29
	Tebuthiuron	16	38 (22–62)	5.2±2.57	0.0 (0.0–2.85)	2	50 (6–100)	24±24	24 (0–48)	1.00	0.42
Brood	Coumaphos	20	100 (61–100)	51.8±12.83	27.5 (5.6–101)	4	100 (27–100)	92.4±32.2	114 (24–139)	1.00	0.33
	Fluvalinate	20	100 (61–100)	844±315	282 (149–930)	4	100 (27–100)	887±418	817 (127–1720)	1.00	0.53

Only pesticides found in 20% or more of samples are reported.

There were neither differences in the mean number of pesticides detected in the wax of CCD-affected colonies (5.92±0.84) compared to control colonies (5.67±0.84; *χ^2^* = 0.001, *P* = 0.97) nor the number of detections in beebread (CCD: 5.09±0.71 vs. control: 5.14±1.14; *χ^2^* = 0.038, *P* = 0.85), brood (CCD: 2.18±0.12 vs. control: 2.07±0.07; *χ^2^* = 0.57, *P* = 0.44), or adult bees (CCD: 4.37±1.73 vs. control: 9.00±3.88; *χ^2^* = 0.89, *P* = 0.34).

Esfenvalerate was more prevalent in the wax of control colonies (32%) when compared to CCD colonies (5%) (Fisher's exact test, *P* = 0.001; Table 10). Mean levels of this product were also higher in both the wax and adult bees from control colonies when compared to CCD colonies (*P* = 0.002 and 0.04, respectively; Table 10). Coumaphos levels in wax, brood, and adult bees were higher in control colonies than in CCD colonies (*P* = 0.009, 0.04, and 0.03, respectively; Table 10).

**Table 10:** The pesticide residue prevalence and load in wax, beebread, brood and adult bees from colonies considered to be normal (control) or affected by CCD (N_t_).

Matrix	Chemical	CCD Colonies		Load		Control Colonies		Load		Prevalence (Fisher's exact test)	Load (Wilcoxon rank sum test)
		N_t_	Prevalence (%) (95%CI)	Mean±S.E.	Median (25th & 75th percentiles)	N_t_	Prevalence (%) (95%CI)	Mean±S.E.	Median (25th & 75th percentiles)	*P*	*P*
Wax	Boscalid	20	40 (24–62)	35.9±11.96	0.0 (0.0–66.0)	15	27 (15–45)	11.59±5.62	0.0 (0.0–0.0)	0.48	0.27
	Chlorothalonil	42	36 (26–49)	22.5±7.53	0.0 (0.0–16.15)	28	21 (14–30)	19.88±17.04	0.0 (0.0–0.0)	0.28	0.16
	Chlorpyrifos	42	73 (53–99)	6.5±1.62	4.1 (0.0–7.52)	28	75 (50–100)	5.83±1.25	5.6 (0.25–7.4)	1	0.68
	Coumaphos	42	100 (72–100)	2645±500	1335 (524–3320)	28	100 (67–100)	5330±1053	4090 (1435–6270)	1	0.0^*^
	Dicofol	42	19 (14–26)	5.3±4.92	0.0 (0.0–0.0)	28	11 (7–16)	0.9±0.58	0.0 (0.0–0.0)	0.51	0.43
	Endosulfan	42	21 (15–28)	2.0±0.94	0.0 (0.0–0.0)	28	25 (17–36)	6.88±4.57	0.0 (0.0–1.2)	0.77	0.67
	Esfenvalerate	42	5 (4–7)	1.4±1.33	0.0 (0.0–0.0)	28	32 (21–46)	1.98±0.84	0.0 (0.0–1.6)	0.00^*^	0.00^*^
	Fluvalinate	42	100 (72–100)	11825±1906	9420 (3067–15275)	28	100 (67–100)	21844±7350	9565 (2570–29675)	1	0.53
	Iprodine	24	21 (13–31)	32.5±15.83	0.0 (0.0–0.0)	14	14 (8–24)	59.5±42.6	0.0 (0.0–0.0)	1	0.75
Beebread	Atrazine	11	36 (18–64)	8.8±5.31	0 (0–4.7)	7	16 (6–33)	5.42±5.42	0.0 (0.0–0.0)	0.60	0.32
	Chlorpyrifos	11	91 (45–100)	2.14±1.03	0.7 (0.3–1.8)	7	86 (35–100)	1.0±0.47	0.7 (0.4–0.8)	1	0.82
	Coumaphos	11	45 (22–81)	24.02±10.73	0 (0–67)	7	57 (23–100)	5.4±2.87	4.2 (0.0–7.2)	1	0.81
	Dicofol	11	18 (9–32)	0.22±0.16	0 (0–0)	7	42 (17–87)	0.29±0.15	0.0 (0.0–0.7)	0.32	0.39
	Endosulfan1	11	27 (12–48)	0.18±0.10	0 (0–0.5)	7	43 (17–89)	0.37±0.18	0.0 (0.0–0.8)	0.63	0.39
	Fenpropathrin	11	54 (27–48)	1.45±0.41	0.8 (0–1.6)	7	57 (23–100)	0.83±0.36	0.8 (0.0–2.0)	1	0.85
	Fluvalinate	11	100 (50–100)	351±235	94 (7.4–339)	7	86 (35–100)	99±42.1	59 (12–193)	0.38	0.75
	Malathion	11	27 (13–48)	0.96±0.70	0 (0–0.9)	7	14 (6–29)	0.51±0.51	0 (0–0)	1	0.57
	Tebuthiuron	11	27 (13–48)	4.69±3.07	0 (0–1.6)	7	57 (23–100)	11.42±7.12	0 (0–27)	0.33	0.19
Brood	Coumaphos	13	100 (53–100)	30.76±11.88	21 (3.9–40.5)	11	100 (50–100)	91.39±18.26	111 (17–137)	1	0.04^*^
	Fluvalinate	13	100 (53–100)	1044±479	279 (135–1043)	11	100 (50–100)	623±197	364 (149–1270)	1	0.67
Adults	Chlorothalonil	9	33 (15–63)	4.56±4.07	0.0 (0.0–2.0)	7	29 (12–60)	0.81±0.53	0.0 (0.0–2.5)	1	0.89
	Chlorpyrifos	9	33 (15–63)	0.37±0.19	0.0 (0.0–1.0)	7	29 (12–60)	0.34±0.22	0.0 (0.0–1.0)	1	0.94
	Coumaphos	9	78 (36–100)	48.3±40.12	7.4 (0.5–22.5)	7	100 (40–100)	57.2±15.53	65 (11–100)	0.48	0.03^*^
	Esfenvalerate	9	43 (20–82)	0±0	0.0 (0.0–0.0)	7	11 (4–23)	3.75±1.77	0.0 (0.0–8.5)	0.26	0.04^*^
	Endosulfan (total)	9	0	0.9±0.9	0.0 (0.0–0.0)	7	29 (12–60)	0.8±0.8	0.0 (0.0–0.0)	0.18	0.92
	Fluvalinate	9	100 (46–100)	1769±814.6	238 (88.5–4540)	7	100 (40–100)	333±215.0	142 (91–185)	1	0.22

Only pesticides found in 20% or more of samples are reported.

### Comparison of mitotypes

Only one of the 98 colonies screened for mitotype was found to be Western European in matrilineal origin. The remaining colonies were all found to be of Eastern European origin. None were positively detected as being African in origin.

## Discussion

This descriptive epidemiological study was initiated to better characterize CCD and compare risk-factor exposure between control and afflicted populations in hopes of identifying factors that cause or contribute to Colony Collapse Disorder. Of the more than 200 variables we quantified in this study, 61 were found with enough frequency to permit meaningful comparisons between populations. None of these measures on its own could distinguish CCD from control colonies. Moreover, no single risk factor was found consistently or sufficiently abundantly in CCD colonies to suggest a single causal agent. Nonetheless, our results help to elucidate this poorly understood affliction of the honey bee colonies and provide insight into the planning of hypothesis-driven research.

CCD apiaries contained more dead and weak colonies than did control apiaries and the distribution of dead and weak colonies in CCD apiaries was not random. Dead and weak colonies were more likely to neighbor each other in CCD apiaries as compared to control apiaries (Table 3), suggesting that an infectious agent or the exposure to a common risk factor may be involved in colony collapse.

While no single pathogen or parasite was found with sufficient frequency to conclude a single organism was involved in CCD, pathogens seem likely to play a critical (albeit secondary) role. CCD colonies generally had higher virus loads and were co-infected with a greater number of disease agents than control colonies. Elevated virus and *Nosema* spp. levels potentially explain the symptoms associated with CCD. One possible way honey bees regulate pathogen and parasite loads within a colony is for infected individuals to emigrate from their hive [69]. This behavior has been proposed to explain the rapid loss of adult populations in colonies collapsing from *N. ceranae* [39]. Whether infected individuals die away from the hive as the result of an evolved response (suicidal pathogen removal [69]) or from a sudden debilitating process by which forager bees cannot return to the hive [39] is irrelevant to understanding how colony collapse can unfold. Premature loss of worker bees does not preclude non-pathogenic causes; recent work has shown that worker bee longevity can be reduced when they are exposed to sub-lethal levels of coumaphos during the larval and pupal stages (Pettis, unpublished). The premature loss of forager bees, the older cohort in a colony, results in younger bees prematurely becoming forager bees [70]. If these replacement bees die at a rate that exceeds the colony's ability to replace them, the result would be rapid depopulation, a reduction in the bee-to-brood ratio, and eventually colony failure.

This study verified initial field observations [1] that there was a difference in the bee-to-brood ratio between CCD-affected populations when compared to controls. If the bees in colonies undergoing CCD collapse are young bees (as field observations suggest), we would expect to find indirect evidence of this in the measures of parasite loads with known associations to bee age. Tracheal mite loads increase as bees age [71], possibly explaining why HBTM incidence and prevalence were higher in control apiaries than in CCD-affected apiaries. Alternatively, HBTM levels may be lower in CCD colonies because infested individuals left the colony.

An unavoidable bias that results from sampling colonies in the midst of collapse is that only surviving bees are collected. These bees, arguably, are the least sick or most fit individuals. Asymmetry is expected to increase when stressful conditions disturb the normal development of insects [72]. In honey bees specifically, increased levels of symmetry correlates to increased fitness [53]. Bees from colonies suffering from CCD were consistently more symmetrical than those from control colonies. It is therefore reasonable to assume that bees surviving in CCD colonies, while young, were the fittest bees, surviving longer than their less-fit sisters. While this assumption needs to be verified experimentally, a comparison of the ranges of FA in populations of bees from CCD colonies versus control colonies provides tacit support to this hypothesis. The lower ranges of FA measures were comparable between CCD and control populations (25^th^ percentile: 1.3 vs. 1.4 for CCD and control colonies, respectively), while the upper range of FA measures was notably higher in control colonies when compared to CCD colonies (75^th^ percentile: 2.2 vs. 1.8, respectively), suggesting that bees in CCD colonies under the most development stress (and with the greatest FA) had left or been removed from colonies before sampling.

Recently, *N. ceranae* was linked to colony losses in Spain [73], and a subsequent study documented how pathogen levels developed over time. In the final stages of collapse, the young bees remaining in the colony became heavily infected with this agent [39]. Our survey found only about half of the colonies sampled, both in CCD and control populations, were infected with *N. ceraneae*, and while some colonies had levels of infection that likely contributed to colony loss, this was not the case for the majority.

In a previous study using subsamples from the same colonies sampled here, IAPV was identified as highly correlated to CCD [3]. This expanded study did not replicate those results. The overall incidence of IAPV reported here was generally lower than found in the prior survey. This result might reflect decreased sensitivity of the assay used here, although prevalence of other viruses generally was comparable to prior results. Alternatively, the discrepancy in findings might reflect unappreciated genetic variation across lineages of IAPV, to the extent that primers poorly matched template cDNA. To minimize this risk, we estimated transcript levels using three published primer pairs for three regions of the genome, and we found broadly concordant results (Tables 6 and 7). As in [3], we treated products for any of the three used primer pairs as evidence for IAPV presence. Finally, the current survey included more colonies and covered a wider geographical range than the previous survey. IAPV shows strong geographical patterns (Evans JD et al., unpublished), and it is expected that surveys for this and other pathogenic viruses will differ across apiaries and regions [74].

The intrinsic bias associated with sampling only surviving (and presumably the least-sick) bees did not prevent us from establishing that workers in CCD colonies were more ill than those in control colonies. Co-infection with both *Nosema* species was 2.6 times greater in CCD colonies when compared to control colonies, and colonies co-infected with 4 or more viruses were 3.7 times more frequent in CCD colonies than in control colonies. While honey bee colonies are commonly infected with one or more pathogens, often without exhibiting overt signs of illness [75], the greater prevalence and abundance of infectious agents in CCD colonies does suggest that either they were exposed to a greater number of pathogens or their ability to fight infection had been compromised.

Several factors are known or suspected to be able to compromise the honey bee immune response. One proposed factor is poor nutrition. In this study, we measured protein content as a surrogate for evidence of poor nutrition in CCD colonies, and these results suggest that nutrition does not play a decisive factor. However, caution is needed in drawing strong inferences from these findings, as nutritional deficiencies may have much more subtle effects on bee development and immunity than can be detected with our methods.

Chronic or sub-lethal exposure to agricultural- or beekeeper-applied pesticides can weaken the honey bee immune system [48], hampering the ability of bees to fight off infection. This study found no evidence that the presence or amount of any individual pesticide occurred more frequently or abundantly in CCD-affected apiaries or colonies. In fact, the opposite was true; two products, esfenvalerate in wax, and coumaphos in wax, brood, and adult bees were found more frequently and at higher levels in control colonies than in CCD colonies.

Esfenvalerate or fenvalerate (racemic form), a pyrethroid insecticide, is considered to be highly toxic to bees [76], but its threat to honey bees is thought to be minimal as it tends to repel them. Exposed forager bees are thought to die in the field before returning to the hive [77], so detection of this product in wax is curious. Finding this product more frequently and at higher levels in control colonies may be spurious, however, similar residue levels in both CCD and control apiaries suggest uniform in-field exposure between populations.

Coumaphos is a product used by beekeepers to control varroa mites. Elevated levels of this product in control apiaries suggest that beekeepers managing those apiaries had more aggressively controlled for this parasitic mite than beekeepers managing CCD apiaries. In addition, control apiaries tended to have higher levels of fluvalinate (*P* = 0.06), another approved acaricide. Regardless of these differences in mite-control compounds, we were unable to detect differences in varroa mite levels in CCD- compared to control apiaries or colonies, suggesting that this mite was not the immediate cause of CCD. This does not necessarily mean that mite infestations have no role in collapse. It is possible that some of the sampled colonies had their mite populations controlled by miticides a few months prior to our sampling. Thus, while mite populations were comparable between the two groups at the time of sampling, there may have been a difference in the mite populations prior to mite treatment applications. Varroa mite parasitism is known to weaken the bees' immune system [78] and facilitate the transmission of viruses to brood and adult bees [79]. Further, high virus levels resulting from high populations of varroa mites are not always immediately suppressed by effective mite control [80]. The potential “legacy” effect of high mite populations in CCD-affected colonies should be the focus of future longitudinal epidemiological studies prior to the categorical dismissal of varroa mites as a causal or contributing agent in CCD.

Coumaphos, an organophosphate, is lipophilic, and so accumulates in wax. Increased levels of the compound in wax have been shown to decrease survivorship of developing queens [81], [82]. Similar results with worker bees have also been recorded (Pettis, unpublished). A quick method to assess larval survival is to quantify the number of empty brood cells in an area of capped brood or, to use the beekeeper colloquial term, brood “spottiness”. We found no evidence that bees from control colonies had a greater frequency of spotty brood than CCD colonies despite the elevated levels of coumaphos in wax in the control colonies. This suggests that bees in control colonies had developed a tolerance to coumaphos exposure. Coumaphos-tolerant bees may be afforded protection through several routes. First, by living on wax comb with elevated miticide levels, varroa mite populations may remain lower than they would in colonies with lower levels of coumaphos residues in their brood nest. However, as coumaphos-resistant mites are widespread in the U.S. [81], this explanation seems unlikely unless coumaphos-resistant mites are less fit than non-resistant mites. Even a small reduction in the reproductive fitness of varroa mites could have a pronounced effect on their population growth and thus their effect on colony health [83]. Second, coumaphos (and/or fluvalinate) tolerance in bees provides cross-resistance to pesticide exposures from other organophosphates and pyrethroids [84] which may be affecting CCD-afflicted bees at sub-lethal doses. Honey bees, as compared to other insects, are notably lacking in detoxification enzymes which provide moderate levels of cross-resistance to pesticides [85]. Any enhancement in these enzyme levels may greatly improve the ability of bees to tolerate the numerous pesticides they encounter in-hive or while foraging.

When unexplained disease outbreaks occur, epidemiologists use descriptive studies to help identify possible cause(s). By definition, descriptive studies are non-hypothesis driven but rather highlight differences between diseased and non-diseased populations in an effort to inform future research.

This descriptive study looked for differences in colony strength, morphometrics, and risk factors in CCD and control colonies. Like all descriptive studies, we cannot make any definitive statement concerning which factors do or do not contribute to or cause CCD. However, our results permit some valuable inferences to be drawn, as the distribution of CCD-infected colonies was not random in infected apiaries and thus the underlying factor is likely contagious or caused by exposure to a common risk factor(s). As no one disease agent was found in all CCD colonies, and because bees derived from CCD colonies were infected with more pathogens then their control colony counterparts, we suspect that while pathogen infection may cause the symptoms of collapse, these infections are secondary and are the result of some other factor or combination of factors that reduce the bees' ability to mitigate infection. As mentioned throughout the text, these inferences must be considered in concert with the limitations and assumptions that are intrinsic to epidemiological studies.

For practical reasons, quantifying most factors in honey bee colonies (e.g., parasite loads, physiological measures, pesticide and pathogen loads) involves testing a sub-sample of colonies in a population. While increasing sample size would obviously result in increased test specificity, this was not always logistically possible. Moreover, our approach assumes that the factor(s) responsible for CCD would occur with high frequency in the affected population. Should this not be the case, our efforts may not have been resolute enough to detect it. Our study also assumes that the factor(s) responsible for CCD were present in the colonies at the time of sample collection, which also may not have been the case. For example, if pollen contaminated with a pesticide were responsible for CCD, contaminated pollen would have been consumed prior to sample collection and thus would not have been detected in the samples collected. Similarly, bees infected with the causative disease agent could have died away from the colony and thus not collected. Finally, Varroa mites or other parasites could have differed among populations prior to sampling, but effective control measures masked these differences at the time of sample collection.

Descriptive studies rely on operational case definitions. The case definition used in this study was applied by experienced bee clinicians using easily observable characteristics [9]. While the application of the case definition may have misdiagnosed colonies, our finding that colony strength measures differed between CCD and control colonies suggests the classification of colonies into affected and non-affected groups was not random. As with other descriptive studies based on case definitions, our findings enable us to propose refining the operational case definition of CCD [8]. In addition to the characteristics of CCD colonies previously described—(1) no dead bees in the colonies or apiary, (2) adult populations rapidly declined leaving brood poorly or completely unattended, and (3) the absence of robbing or kleptoparasitism in collapsed colonies—we now propose that the operational case definition for CCD include (4) at the time of collapse, varroa mite and nosema populations are not at levels known to cause economic injury or population decline. This additional characteristic should assist in distinguishing diminishing populations associated with elevated levels of varroa mites (and virus) [86] and *N. ceranae* [39] from collapsing populations associated with CCD.

The primary aim of descriptive studies is to help narrow future efforts that attempt to identify the cause of disease. This study suggests that future, longitudinal studies should focus on monitoring parasite (varroa mite), pathogen, and pesticide loads while quantifying pesticide tolerance in study populations. More specific studies that investigate potential interactions among pesticides and pathogen loads are also warranted.

This is the first descriptive epizootiological survey of honey bee colonies that provides evidence that the condition known as CCD is consistent with a contagious condition or reflective of common risk factors within apiaries Of the 61 variables quantified (including adult bee physiology, pathogen loads, and pesticide levels), no single factor was found with enough consistency to suggest one causal agent. Bees in CCD colonies had higher pathogen loads and were co-infected with more pathogens than control populations, suggesting either greater pathogen exposure or reduced defenses in CCD bees. Levels of the miticide coumaphos were higher in control populations than CCD-affected populations. Potentially important areas for future hypothesis-driven research, including the possible legacy effect of mite parasitism and role of honey bee resistance to pesticides, are highlighted.
